# Nutritional status and dietary diversity of orphan and non – orphan children under five years: a comparative study in the Brong Ahafo region of Ghana

**DOI:** 10.1186/s40795-018-0240-0

**Published:** 2018-07-30

**Authors:** Zakari Ali, Nurudeen Abu, Isaac Aning Ankamah, Esther Abena Gyinde, Alimatu Sadia Seidu, Abdul-Razak Abizari

**Affiliations:** Department of Nutritional Sciences, School of Allied Health Sciences, University for Development Studies, P O Box 1883, Tamale, Ghana

**Keywords:** Nutritional status, Dietary diversity, Orphans, Orphanages, Children, Ghana

## Abstract

**Background:**

Undernutrition in children under 5 years is a major risk factor to child deaths and is related to impaired cognitive development and lower school performance. Underprivileged children such as orphans are at particularly high risk of undernutrition. Little is however known about the nutritional status and dietary diversity of orphan children in Ghana. This study therefore compared the nutritional status and dietary diversity of orphan and non- orphan children.

**Methods:**

An analytical cross – sectional study design was used. Two hundred and forty-six children (123 non- orphan and 123 orphans) were sampled from households (non - orphans) and four orphanages (orphans). Maternal / caregiver and child socio-demographic characteristics and dietary diversity of children was assessed with a semi – structured questionnaire. We measured anthropometric characteristics of children. Stunting, wasting and underweight in children was classified using Height – for – age Z – scores (HAZ), Weight – for – height Z – scores (WHZ) and Weight – for – age Z – scores (WAZ) respectively. Bivariate and multivariate analyses were used to compare the nutritional status and dietary diversity of orphan and non- orphan children.

**Results:**

Majority of the children were male (52.4%). The prevalence of stunting, wasting and underweight was 17.9, 5.3 and 7.7% respectively for all children. There was no difference in the prevalence of stunting (17.1% vs 18.7%) (*p* = 0. 74), wasting (4.9% vs 5.7%) (*p* = 0.78) and underweight (7.3% vs 8.1%) (*p* = 0.81) among non – orphans and orphans. There was also no difference in mean HAZ (*p* = 0.52), WHZ (*p* = 0.27) and WAZ (*p* = 0.12) of non- orphan and orphan children. However, orphans had higher mean dietary diversity score (*p* <  0.001) and minimum dietary diversity (p <  0.001) than non – orphans. After controlling for potential confounders, non – orphans were 7.491 times more likely to have a low dietary diversity [AOR = 7.491; 95% CI (1.851–30.320); *p* = 0.005] compared to orphans.

**Conclusion:**

Present study data show no significant difference in the anthropometric status of orphan and non – orphan children. Orphans were more likely to receive a diversified diet than non – orphans.

## Background

Undernutrition in children under 5 years is a major risk factor to child deaths with an estimated 2.2 million deaths associated with it [1]. Undernutrition in children especially stunting has been linked with lower human capital [2], impaired cognitive development and lower performance in school [3]. Sub – Saharan Africa and south Asia are home to majority of the world’s chronically undernourished children than elsewhere in the world [4].

In Ghana, child undernutrition is a major public health problem. The prevalence of stunting in some regions have reached as high as 33% [5].

The number of children who are left orphaned in the world due to loss of parents has increased in recent years. An estimated 153 million children in the world are orphans [6], with more than one in seven children orphaned in sub- Saharan Africa [7]. The orphan child population in Ghana has been estimated to be over 1.1 million [8].

HIV/AIDS infection is by far the major culprit in leaving children orphaned in most cases [9]. Children who have lost one or both parents, or being abandoned by parents are said to be orphans, and includes children below 18 years of age [10]. The extended family is an important care provider to orphans in sub – Saharan Africa [11], while a majority of them are also institutionalized in orphanages [12].

Being an orphan may make children more vulnerable to undernutrition [13–15], as maternal and paternal level factors, and household food availability which are linked to child nutritional well – being are more likely to be inadequate [16]. However, studies have reported mixed results about how the nutritional status of orphans compare with their counterpart non – orphans [17, 18], and dietary diversity of orphans have received little study. There is scarcity of data on the nutritional status and dietary diversity of orphans in Ghana and the Brong Ahafo region in particular. Understanding the disparities in nutritional status and dietary diversity of orphan and non – orphan children could be useful to nutrition policy makers and intervention planners to target children who are more vulnerable to undernutrition and inadequate dietary intake. Present study therefore aimed to compare the nutritional status and dietary diversity of orphan and non- orphan children.

## Methods

### Study area

This study was conducted from December 2015 to January 2016 in the Sunyani municipality in the Brong Ahafo region of Ghana. The Sunyani municipal is the administrative seat of the region and one of the 27 districts in the Brong Ahafo region. The municipality has a total land area of 506.7 km^2^. The municipality shares boundaries with the Sunyani West district to the north; Dormaa East district to the west; Asutifi district to the south and to the east by Tano North district. According to the 2010 population and housing census, the total population of the municipality is 123,224 with 50.1% of them being females and 49.9%. Children aged 0–4 years make up 11.1% of the population. The municipality has a total age dependency ratio of 54.0 and a fertility rate of 2.6.

More than three in ten (37.6%) of women aged 12 years and above are married while 47.2% have never married. Other forms of marital status of women in the region include consensual unions (10.0%), widowed (3.1%), divorced (1.0%) and separated (1.0%) [19].

### Study design, population and sampling

Present study used a cross sectional study design. The target population was orphans and non- orphans aged 0–59 months. The orphans were recruited from orphanages and non-orphans from households.

A required sample size of 246 children was calculated from 19.3% [20] prevalence of chronic undernutrition in children under-five in the Brong Ahafo region and using a 5% margin of error and a 95% confidence interval (CI) and a none response rate of 3%.

One hundred and twenty-three orphans from orphanages and 123 non – orphans in households were selected for this study. The orphans were drawn from four orphanages in the Sunyani municipality, located at Yawhima (Yawhima children’s home), Abesim (Alifa, the kid’s shelter), Baakoniaba (Hanuka children’s home) and Nkrankrom (Home for kids and aged) communities. The non – orphans were also drawn from household in these communities.

Orphans were selected using simple random sampling technique while the modified random walk was used to select non – orphan children from communities. For the orphans, a list comprising children aged 0–59 months was compiled for each orphanage and numbered consecutively. A maximum of 31 orphans were then drawn at random from each list using Excel generated random numbers. For the non- orphans, households were visited to identify eligible children following the modified random walk methodology as reliable household lists were not available in the communities. Important land marks including market centres, churches, mosques, schools, chief palace, clinics and information centres in the communities were identified and listed. A random selection of one of these key land marks was then made and the first house closest to the landmark was chosen as the first household from which children within the target age group were selected. Where a target group is not available in the first house closest to the landmark, the next house close to the landmark was visited. A child in the households qualified for inclusion as non – orphan upon verification that none of the child’s parents were dead and that the child is not abandoned by parents. In households with more than one eligible child, the index child (youngest) was chosen.

For orphans aged 6–59 months, a minimum of 4 months stay in an orphanage was required to be eligible for inclusion in this study. There was no specified duration of stay for orphans below 6 months of age in orphanages, however those with higher stay duration were included in the sampling frame to be subjected to the random selection.

### Data collection procedure

Pretested structured questionnaires were used to collect data in face – to – face interviews in households and orphanages. Primary caregivers in orphanages were interviewed on behalf of selected children. In households in the case of non -orphan children, mothers were interviewed on behalf of their selected children. The questionnaire elicited information on socio-demographic characteristics of mothers/primary caregivers and children and dietary intake of the children.

### Nutritional status assessment

Length/height, weight and age of children were measured based upon which anthropometric z-scores were generated to classify nutritional status of children. All children were measured in only underwear or light clothing appropriate for the situation during measurements. The measurements were taken following WHO standard procedures [21]. Date of birth information of children was taken from child health records booklets and birth certificates. In some cases, date of birth information of orphans was obtained from child registers in orphanages.

### Assessment of dietary diversity

The dietary quality of children was assessed using dietary diversity score. The validated seven food groups by WHO were used to assess dietary diversity of children [22].

Structured 24-h dietary recall was used to assess foods consumed by children. Mothers (in the case of non – orphans) were asked to recall the number of times in the past 24 h a child had received anything to eat aside from breast-milk, including meals and snacks. In the case of orphans, primary caregivers were interviewed and daily meal menus were also examined for consistency and meal composition. Children were considered to have eaten from a particular food group when any quantity of food in that food group is consumed except when such foods are used as condiments [23]. The dietary diversity score ranged from 0 to 7. A child had a score of 0 if none of the food groups was consumed and seven if all the food groups were consumed.

The WHO defines minimum dietary diversity as the proportion of children aged 6–23 months who received foods from at least four out of seven food groups [23]. In this study however, the minimum dietary diversity indicator was calculated for children 6–59 months who received foods from at least four out of the seven food groups.

Below are the seven food groups used in defining minimum dietary diversity indicator of children: (i) grains, roots and tubers; (ii) legumes and nuts; (iii) dairy products; (iv) flesh foods (meats/fish/poultry) (v) eggs (fowl/guinea fowl/duck eggs) (vi) vitamin A rich fruits and vegetables; and (vii) other fruits and vegetables.

### Statistical analysis

Data analyses was performed with the Statistical Package for Social Sciences (SPSS), version 21.0 for Windows. Categorical data were presented as frequencies and percentages while continuous data were presented as means and standard deviations.

WHO Anthro software (version 3.2.2) was used to convert length/height, weight and age measurements of children to Height- for- age Z- scores (HAZ), weight- for –height Z- scores (WHZ) and weight- for- age (WAZ) which were used to classify stunting, wasting and underweight respectively. Undernutrition was defined as Z- scores below − 2 standard deviations below the median of the WHO reference population. The 2006 World Health Organization (WHO) growth standards were used to generate the Z-scores (HAZ, WHZ and WAZ) and subsequent calculation of the prevalence of stunting, wasting and underweight.

Chi square test was used to determine the association between child nutritional status (stunting, wasting and underweight) and their parenting status (orphan or non- orphan) and the minimum dietary diversity of children. Student t – test was also used to compare mean anthropometric z- scores (HAZ, WHZ and WAZ) and dietary diversity scores of orphan and non – orphan children. Regression analysis was performed for differences that were significant in bivariate Chi square analysis. We used multivariate logistic regression to establish the independent contribution of child status (orphan/non- orphan) to meeting the minimum dietary diversity while controlling for potential confounders. Results were considered significant at *p* <  0.05.

## Results

### Socio – Demographic characteristics

The majority of the children were male (52.4%). The sex distribution of orphan and non- orphan children was not similar. For example, while 56.9% of orphan children were male, only 48% of non – orphan children were male. The children had mean age ± SD of 28.4 ± 15.70 months. Most of the children were aged between 24 and 35 months (25.6%).

Mothers / caregivers had mean age ± SD of 37.9 ± 11.4 years. Caregivers at orphanages were generally older than non – orphan child mothers (46.2 ± 8.9 versus 29.6 ± 6.4, respectively). Overall, majority of mothers / care givers were more than 35 years old (56.9%), were currently married (61%), had attained at least senior high school education (SHS) (61.8%) and belonged to the Akan ethnic group (61.4%). Table 1 presents the socio – demographic characteristics of children and mothers / caregivers.

**Table 1:** Socio- demographic characteristics

Characteristics	Frequency (%) / mean ± SD		All
	Non - orphan	Orphan	
Child characteristics			
Sex			
Male	59 (48)	70 (56.9)	129 (52.4)
Female	64 (52)	53 (43.1)	117 (47.6)
Mean age	26 ± 16.73	30.8 ± 14.3	28.4 ± 15.70
Age group (months)			
0–5	19 (15.4)	6 (4.9)	25 (10.2)
6–11	10 (8.1)	5 (4.1)	15 (6.1)
12–23	31 (25.2)	27 (22)	58 (23.6)
24–35	24 (19.5)	39 (31.7)	63 (25.6)
36–47	18 (14.6)	27 (22)	45 (18.3)
48–59	21 (17.1)	19 (15.4)	40 (16.3)
Maternal/caregiver characteristics			
Mean age	29.6 ± 6.4	46.2 ± 8.9	37.9 ± 11.4
Age group (years)			
≤ 35	106 (86.2)	0 (0)	106 (43.1)
More than 35	17 (13.8)	123 (100)	140 (56.9)
Marital status			
Currently married	96 (78)	54 (43.9)	150 (61)
Currently unmarried	27 (22)	69 (56.1)	96 (39)
Ethnicity			
Akan	76 (62.3)	75 (61)	151 (61.4)
Others	47 (38.2)	48 (39)	95 (38.6)
Level of education			
None	21 (17.1)	0 (0)	21 (8.5)
At least senior high school	98 (79.7)	54 (43.9)	152 (61.8)
Tertiary	4 (3.3)	69 (56.1)	73 (29.7)

### Nutritional status and dietary diversity of non – Orphan and orphan children

Table 2 presents the nutritional status and dietary diversity of non- orphan and orphan children. The nutritional indices of the entire study population were below the WHO standard population, indicated by the negative z – scores. The prevalence of stunting, wasting and underweight among children was 17.9, 5.3 and 7.7% respectively. There was little variation in the prevalence of undernutrition among non-orphan and orphan children. For instance, while 17.1% of non – orphans were stunted, 18.7% of orphans were stunted. This was similarly observed for wasting (4.9% versus 5.7%, respectively for non- orphan and orphan children) and underweight (7.3% versus 8.1%, respectively for non- orphan and orphan children).

**Table 2:** Nutritional status and dietary diversity of non- orphan and orphan children

Item	Frequency(%)/ mean ± SD		
	Non- orphan	Orphan	All
Z- scores			
Mean height-for age-z-score (HAZ)	−1.05 ± 0.86	−0.97 ± 1.01	−1.01 ± 0.94
Mean weight-for-height-z-score (WHZ)	−0.86 ± 0.59	−0.76 ± 0.82	−0.81 ± 0.72
Mean weight-for-age-z-score (WAZ)	−1.20 ± 0.61	−1.06 ± 0.81	−1.13 ± 0.72
Prevalence of undernutrition			
Stunting (HAZ < −2)	21 (17.1)	23 (18.7)	44 (17.9)
Wasting (WHZ < −2)	6 (4.9)	7 (5.7)	13 (5.3)
Underweight (WAZ < −2)	9 (7.3)	10 (8.1)	19 (7.3)
Food groups consumed			
^a^Grains, roots and tubers	99 (95.2)	117 (100)	216 (97.7)
^a^Legumes and nuts	49 (47.1)	104 (88.9)	153 (69.2)
^a^Dairy products;	28(26.9)	116 (99.1)	144 (65.2)
^a^Flesh foods (meats/fish/poultry)	89 (85.6)	114 (97.4)	203 (91.9)
^a^Eggs	14 (13.5)	33 (28.2)	47 (21.3)
^a^Vitamin A rich fruits and vegetables	85 (81.7)	115 (98.3)	200 (90.5)
^a^Other fruits and vegetables	91 (87.5)	112 (95.7)	203 (91.9)
Dietary diversity			
^a^Mean dietary diversity score (dds)	4.37 ± 1.3	6.1 ± 0.7	5.3 ± 1.4
^a^Minimum dietary diversity (mdd)	86 (82.7)	114 (97.4)	200 (90.5)

^a^Calculated based on children aged 6–59 months (104 non- orphans and 117 orphans)

More than nine in ten children consumed from the following food groups; grains, roots and tubers (97.7%), flesh foods (91.9%), vitamin A rich fruits and vegetable (90.5%), and other fruits and vegetables (91.9%) in the day preceding the study. More than two- thirds of children consumed legumes (69.2%) while more than six in ten children consumed dairy products in the previous day. Eggs (21.3%) was the food group least consumed by children. The consumption of food groups varied greatly among non- orphan and orphan children with orphan children generally having higher intakes. For example, while only 26.9% of non- orphans consumed dairy products, 99.1% of orphans consumed from this food group. Similarly, while 47.1% of non- orphans consumed legumes, 88.9% of orphans consumed foods from it.

The mean dietary diversity score of children was 5.3 ± 1.4. This was not similar among non- orphan and orphan children (4.37 ± 1.3 versus 6.1 ± 0.7 respectively). A little above 90 % of children consumed from at least four food groups (90.5%) in the previous day. The minimum dietary diversity of orphans (97.4%) was a little higher than non- orphans (82.7%).

### Comparison of nutritional status and dietary diversity of orphans and non- orphans

Bivariate Chi square analysis of study data revealed no significant difference in the prevalence of stunting (*p* = 0.74), wasting (*p* = 0.78) and underweight (*p* = 0.81) among non- orphan and orphan children. Minimum dietary diversity was however, significantly higher among orphans (*p* <  0.001) (Table 3).

**Table 3:** Comparison of undernutrition prevalence and minimum dietary diversity of children

Item	Child status		*p* - value
	Non- orphan	Orphan	
Stunted			
Yes	21 (17.1%)	23 (18.7%)	0.74
No	102 (82.9%)	100 (81.3%)	
Wasted			
Yes	6 (4.9%)	7 (5.7%)	0.78
No	117 (95.1%)	116 (94.3%)	
Underweight			
Yes	9 (7.3%)	10 (8.1%)	0.81
No	114 (92.7%)	113 (91.9%)	
Minimum dietary diversity			
Less than 4 food groups	18 (17.3%)	3 (2.6%)	< 0.001
At least 4 food groups	86 (82.7%)	114 (97.4%)	

Further, there was no significant difference in mean z- scores of non- orphan and orphan children. Mean Height- for- age z- scores (*p* = 0.522), Weight- for- height z- scores (*p* = 0.272) and Weight- for- age z-scores (*p* = 0.122) were not different among non – orphan children and orphan children. Orphan children however had higher mean dietary diversity scores than non- orphan children (*p* <  0.001) (Table 4).

**Table 4:** Comparison of mean anthropometric z- scores and dietary diversity scores of children

Indicator	N	Mean	SD	F	*p* - value
Height –for –age z- score (HAZ)					
Non- orphan	123	−1.0498	0.863	2.937	0.522
Orphan	123	−0.9731	1.010		
Weight- for- height z- score (WHZ)					
Non -orphan	123	−0.857	0.598	5.94	0.272
Orphan	123	−0.756	0.823		
Weight- for –age z – score (WAZ)					
Non- orphan	123	−1.203	0.610	5.37	0.122
Orphan	123	−1.062	0.807		
Dietary diversity score (dds)					
Non- orphan	104	4.375	1.345	45.29	< 0.001
Orphan	117	6.077	0.745		

Binary logistic regression analysis revealed that non – orphans were 7.491 times more likely to have low dietary diversity compared to orphans [AOR = 7.491; 95% CI (1.851–30.320); *p* = 0.005] after controlling for sex of child and age of child (Table 5).

**Table 5:** Binary logistic regression analysis of determinants of low dietary diversity among children

	AOR	*p* - value	95% C.I. for AOR	
			Lower	Upper
Sex				
Male	1			
Female	1.932	0.252	0.626	5.962
Child status				
Orphan	1			
Non - orphan	7.491	0.005	1.851	30.320
Age group of child (months)		< 0.001		
6–11	1			
12–23	0.018	< 0.001	0.004	0.088
24–59	0.097	0.002	0.023	0.412
Constant	0.280	0.122		

## Discussion

Present study sought to compare the nutritional status and dietary diversity of orphans and non- orphans under 5 years in the Brong Ahafo region of Ghana. The main finding was that there was no significant difference in the nutritional status of orphans in orphanages and non- orphans in households. Our data also show that orphans were more likely to receive a more diversified diet than non- orphans.

The prevalence of child undernutrition as measured by stunting, wasting and underweight among orphan and non – orphan children in this study were consistent with the regional prevalence reported by the demographic and health survey of Ghana [5].

Previous studies have reported mixed findings about the nutritional status of orphans and non- orphans with some reporting poorer nutritional outcomes of orphans while others find no difference. For example, wasting and stunting among orphan Palestinian children was reported to be higher than national prevalence [17]. A study among orphans in an orphanage in Ghana found poor nutritional status of orphans in the Northern region [24]. In another study among orphans under 6 years in Kenya, weight – for – height z – scores were significantly lower among orphans compared to non – orphans [15]. In addition, data from Malawi show poorer nutritional outcomes among orphans in orphanages than non-orphans [25]. The lack of significant difference in the nutritional status of orphan and non-orphan children in present study is consistent with earlier findings. For instance, the risk of child undernutrition was not significantly different between orphan and non- orphan children in Kenya [18]. There was also no significant difference in nutritional status of orphan and non- orphan Luo children [26]. Data from China did not also show significant difference in the nutritional status of orphans and non-orphans [27]. As we find no significant difference in the nutritional statuses of orphan and non- orphan children, efforts aimed at reducing undernutrition among children under 5 years should target both children in orphanages and households with equal importance.

Dietary diversity is shown to be a good indicator of micronutrient adequacy [28] and adequate nutritional status of children [29]. Our data show that, orphans were more likely to receive a diversified diet composed of at least four food groups compared to non – orphans. The significantly higher dietary diversity scores of orphans may indicate better micronutrient intake and may explain the comparable nutritional status of orphans to non – orphans observed in present study. Adequate micronutrient intake among orphans in orphanages has been reported in Nigeria where orphans had adequate intake of iron, calcium, thiamine, and riboflavin [30]. A study among adolescent orphans in a Ghanaian orphanage also found acceptable iodine intake levels [12].

Present study found that orphanages had better daily meal menus which in most of the cases were well followed. Our data therefore disagree with a study among orphanages in India which reported poor menu planning [31]. Our findings highlight the importance of good menu planning in the achievement of adequate dietary diversity among orphans in orphanages.

Among the seven food groups used to assess dietary diversity of orphans and non- orphans, the orphans consumed two food groups which non- orphans did not consume as much. These food groups were legumes and nuts, and dairy products. Most orphanages served beans from the legumes and nuts food group a number of times in the day and in different forms. Beans was commonly combined with rice as rice and beans (locally called “Waakye”) and also used in preparation of stews served with plain rice and boiled yam. These foods were served at different eating moments of the day. Non- orphans in this study seldom consumed beans, they rather consumed groundnuts, which was in few cases prepared as groundnut soup and which is not consumed as regular. The daily meal menus for the orphans included breakfast, lunch and dinner with snacks in some cases. The breakfast of most orphanages in most days incorporated dairy products, mainly powdered milk which increased the consumption of this food group. Households in the communities however, did not follow any planned meal menus and a common breakfast for children was made from fermented corn dough (locally called “koko”) which is normally not served with dairy products. This could explain the consumption of less dairy products among non – orphans. The higher dietary diversity among orphans relative to non- orphans is therefore not surprising and may be explained by the higher intake of legumes and dairy products among the orphans. It is therefore important for mothers in households to increase the inclusion of foods from legumes and nuts and dairy products food groups in complementary foods given to their children. Nutrition education and other interventions aimed at increasing the dietary quality of children should consider ways that make the incorporation of these foods in the diet of children easy for mothers.

It is important to note some limitations of this study. We relied on the memory of mothers and primary care givers for dietary intake of children which may not entirely be correct. However, as index children were chosen in the case of non- orphans, mothers were more likely to remember the food consumption of these children than older children who may eat out of home. For orphans, daily meal menus were checked for consistency of the recalled meals provided by primary caregivers. In spite of these limitations, present study has shed ample light on the nutritional situation and dietary diversity of orphan and non – orphan children in Ghana.

## Conclusion

Present study data show no significant difference in the anthropometric status of orphan and non – orphan children. Orphans were more likely to receive a diversified diet than their counterpart non – orphans.

## Abbreviations

CI: Confidence interval
HAZ: Height-for-age Z-score
SD: Standard deviation
WAZ: Weight-for-age Z-score
WHO: World Health Organization
WHZ: Weight-for-height Z-score
